# Imputation strategies for missing binary outcomes in cluster randomized trials

**DOI:** 10.1186/1471-2288-11-18

**Published:** 2011-02-16

**Authors:** Jinhui Ma, Noori Akhtar-Danesh, Lisa Dolovich, Lehana Thabane

**Affiliations:** 1 Department of Clinical Epidemiology & Biostatistics, McMaster University, Hamilton, ON, Canada; 2 Biostatistics Unit, St Joseph's Healthcare Hamilton, Hamilton, ON, Canada; 3 School of Nursing, McMaster University, Hamilton, ON, Canada; 4 Department of Family Medicine, McMaster University, Hamilton, ON, Canada; 5 Centre for Evaluation of Medicines, St Joseph's Healthcare Hamilton, ON, Canada; 6 Population Health Research Institute, Hamilton Health Sciences, Hamilton, ON, Canada

## Abstract

**Background:**

Attrition, which leads to missing data, is a common problem in cluster randomized trials (CRTs), where groups of patients rather than individuals are randomized. Standard multiple imputation (MI) strategies may not be appropriate to impute missing data from CRTs since they assume independent data. In this paper, under the assumption of missing completely at random and covariate dependent missing, we compared six MI strategies which account for the intra-cluster correlation for missing binary outcomes in CRTs with the standard imputation strategies and complete case analysis approach using a simulation study.

**Method:**

We considered three within-cluster and three across-cluster MI strategies for missing binary outcomes in CRTs. The three within-cluster MI strategies are logistic regression method, propensity score method, and Markov chain Monte Carlo (MCMC) method, which apply standard MI strategies within each cluster. The three across-cluster MI strategies are propensity score method, random-effects (RE) logistic regression approach, and logistic regression with cluster as a fixed effect. Based on the community hypertension assessment trial (CHAT) which has complete data, we designed a simulation study to investigate the performance of above MI strategies.

**Results:**

The estimated treatment effect and its 95% confidence interval (CI) from generalized estimating equations (GEE) model based on the CHAT complete dataset are 1.14 (0.76 1.70). When 30% of binary outcome are missing completely at random, a simulation study shows that the estimated treatment effects and the corresponding 95% CIs from GEE model are 1.15 (0.76 1.75) if complete case analysis is used, 1.12 (0.72 1.73) if within-cluster MCMC method is used, 1.21 (0.80 1.81) if across-cluster RE logistic regression is used, and 1.16 (0.82 1.64) if standard logistic regression which does not account for clustering is used.

**Conclusion:**

When the percentage of missing data is low or intra-cluster correlation coefficient is small, different approaches for handling missing binary outcome data generate quite similar results. When the percentage of missing data is large, standard MI strategies, which do not take into account the intra-cluster correlation, underestimate the variance of the treatment effect. Within-cluster and across-cluster MI strategies (except for random-effects logistic regression MI strategy), which take the intra-cluster correlation into account, seem to be more appropriate to handle the missing outcome from CRTs. Under the same imputation strategy and percentage of missingness, the estimates of the treatment effect from GEE and RE logistic regression models are similar.

## 1. Introduction

Cluster randomized trials (CRTs), where groups of participants rather than individuals are randomized, are increasingly being used in health promotion and health services research [1]. When participants have to be managed within the same setting, such as hospital, community, or family physician practice, this randomization strategy is usually adopted to minimize the potential treatment "contamination" between intervention and control participants. It is also used when individual level randomization may be inappropriate, unethical, or infeasible [2]. The main consequence of the cluster-randomized design is that participants can not be assumed independent due to the similarity of participants from the same cluster. This similarity is quantified by the intra-cluster correlation coefficient [ICC] *ρ*. Considering the two components of the variation in the outcome, between-cluster and intra-cluster variations, *ρ *may be interpreted as the proportion of overall variation in outcome that can be explained by the between-cluster variation [3]. It may also be interpreted as the correlation between the outcomes for any two participants in the same cluster. It has been well established that failing to account for the intra-cluster correlation in the analysis can increase the chance of obtaining statistically significant but spurious findings [4].

The risk of attrition may be very high in some CRTs due to the lack of direct contact with individual participants and lengthy follow-up [5]. In addition to missing individuals, the entire clusters may be missing, which further complicates the handling of missing data in CRTs. The impact of missing data on the results of statistical analysis depends on the mechanism which caused the data to be missing and the way that it is handled. The default approach in dealing with this problem is to use complete case analysis (also called listwise deletion), i.e. exclude the participants with missing data from the analysis. Though this approach is easy to use and is the default option in most statistical packages, it may substantially weaken the statistical power of the trial and may also lead to biased results depending on the mechanism of the missing data.

Generally, the nature or type of missingness can fit into four categories: missing completely at random (MCAR), missing at random (MAR), covariate dependent (CD) missing, and missing not at random (MNAR) [6]. Understanding these categories is important since the solutions may vary depending on the nature of missingness. MCAR means that the missing data mechanism, i.e. the probability of missing, does not depend on the observed or unobserved data. Both MAR and CD mechanisms indicate that causes of missing data are unrelated to the missing values, but may be related to the observed values. In the context of longitudinal data when serial measurements are taken for each individual, MAR means that the probability of a missing response at a particular visit is related to either observed responses at previous visits or covariates, whereas CD missing - a special case of MAR - means that the probability of a missing response is dependent only upon covariates. MNAR means that the probability of missing data depends on the unobserved data. It commonly occurs when people drop out of the study due to poor or good health outcomes. A key distinction between these categories is that MNAR is non-ignorable while the other three categories (i.e., MCAR, CD, or MAR) are ignorable [7]. Under the circumstances of ignorable missingness, imputation strategies such as mean imputation, hot deck, last-observation carried forward, or multiple imputation (MI) - which substitute each missing value to one or multiple plausible values - can produce a complete dataset that is not adversely biased [8,9]. Non-ignorable missing data are more challenging and require a different approach [10].

Two main approaches in handling missing outcomes are likelihood based analyses and imputation [10]. In this paper, we focus on MI strategies, which take into account the variability or uncertainty of the missing data, to impute the missing binary outcome in CRTs. Under the assumption of MAR, MI strategies replace each missing value with a set of plausible values to create multiple imputed datasets - usually varying in number from 3 to 10 [11]. These multiple imputed datasets are analyzed by using standard procedures for complete data. Results from the imputed datasets are then combined for inference to generate the final result. Standard MI procedures are available in many standard statistical software packages such as SAS (Cary, NC), SPSS (Chicago IL), and STATA (College Station, TX). However, these procedures assume observations are independent and may not be suitable for CRTs since they do not take into account the intra-cluster correlation.

To the best of our knowledge, limited investigation has been done on the imputation strategies for missing binary outcomes or categorical outcomes in CRTs. Yi and Cook reported marginal methods for missing longitudinal data from clustered design [12]. Hunsberger *et al*. [13] described three strategies for continuous missing data in CRTs: 1) multiple imputation procedure in which the missing values are replaced with re-sampled values from the observed data; 2) a median procedure based on the Wilcoxon rank sum test assigning the missing data in the intervention group with the worst ranks; 3) multiple imputation procedure in which the missing values are replaced by the predicted values from a regression equation. Nixon *et al*. [14] presented strategies of imputing missing end points from a surrogate. In the analysis of a continuous outcome from the Community Intervention Trial for Smoking Cessation (COMMIT), Green *et al *stratified individual participants into groups that were more homogeneous with respect to the predicted outcome. Within each stratum, they imputed the missing outcome using the observed data [15,16]. Taljaard *et al *[17] compared several different imputation strategies for missing continuous outcomes in CRTs under the assumption of missing completely at random. These strategies include cluster mean imputation, within-cluster MI using Approximate Bayesian Bootstrap (ABB) method, pooled MI using ABB method, standard regression MI, and mixed-effects regression MI. As pointed out by Kenward *et al *that if a substantive model, such as generalized linear mixed model, is to be used which reflects the data structure, it is important that the imputation model also reflects this structure [18].

The objectives of this paper are to: i) investigate the performance of various imputation strategies for missing binary outcomes in CRTs under different percentages of missingness, assuming a mechanism of missing completely at random or covariate dependent missing; ii) compare the agreement between the complete dataset and the imputed datasets obtained from different imputation strategies; iii) compare the robustness of the results under two commonly used statistical analysis methods: the generalized estimating equations (GEE), and random-effects (RE) logistic regression, under different imputation strategies.

## 2. Methods

In this paper, we consider three within-cluster and three across-cluster MI strategies for missing binary outcomes in CRTs. The three within-cluster MI strategies are logistic regression method, propensity score method, and MCMC method, which are standard MI strategies conducted within each cluster. The three across-cluster MI strategies are propensity score, random-effects logistic regression method, and logistic regression with cluster as a fixed effect. Based on the complete dataset from the community hypertension assessment trial (CHAT), we conducted a simulation study to investigate the performance of the above MI strategies. We used Kappa statistics to compare the agreement between the imputed datasets and the complete dataset. We also used the estimated treatment effects obtained from the GEE and RE logistic regression model [19] to assess the robustness of the results under different percentages of missing binary outcome under the assumption of MCAR and CD missing.

### 2.1. Complete case analysis

Using this approach, only the patients with completed data are included for analysis, while patients with missing data are excluded. When the data are MCAR, the complete case analysis approach, using either likelihood-based analysis such as RE logistic regression, or the marginal model such as GEE approach, is valid for analyzing binary outcome from CRTs since the missing data mechanism is independent of the outcome. When the data are CD missing, both RE logistic regression and GEE approach are valid if the known covariates associated with the missing data mechanism are adjusted for. It can be implemented using GENMOD and NLMIXED procedure in SAS.

### 2.2. Standard multiple imputation

Assuming the observations are independent, we can apply the standard MI procedures provided by any standard statistical software such as SAS. Three widely used MI methods are predictive model method (logistic regression method for binary data), propensity score method, and MCMC method [20]. In general, both propensity score method and MCMC method are recommended for the imputation of continuous variable [21]. A dataset is said to have a monotone missing pattern when a measurement *Y*_*j *_is missing for an individual implies that all subsequent measurements *Y*_*k*_, *k *>*j*, are all missing for the individual. When the data are missing in the monotone missing pattern, any of the parametric predictive model and the nonparametric method that uses propensity scores or MCMC method is appropriate [21]. For an arbitrary missing data patterns, a MCMC method that assumes multivariate normality can be used [10]. These MI strategies are implemented using MI, MIANALYZE, GENMOD, and NLMIXED procedures in SAS separately for each intervention group.

#### 2.2.1. Logistic regression method

In this approach a logistic regression model is fitted using the observed outcome and covariates [21]. Based on the parameter estimates and the associated covariance matrix, the posterior predictive distribution of the parameters can be constructed. A new logistic regression model is then simulated from the posterior predictive distribution of the parameters and is used to impute the missing values.

#### 2.2.2. Propensity score method

The propensity score is the conditional probability of being missing given the observed data. It can be estimated by the means of logistic regression model with a binary outcome indicating whether the data are missing or not. The observations are then stratified into a number of strata based on these propensity scores. The ABB procedure [22] is then applied to each stratum. The ABB imputation first draws with replacement from the observed data to create a new dataset, which is a nonparametric analogue of drawing parameters from the posterior predictive distribution of the parameters, and then randomly draw imputed values with replacement from the new dataset.

#### 2.2.3. Markov chain Monte Carlo method

Using MCMC method pseudo random samples are drawn from a target probability distribution [21]. The target distribution is the joint conditional distribution of *Y*_*mis *_and *θ *given Y_*obs *_when missing data have a non-monotone pattern, where Y_*mis *_and Y_*obs *_represent the missing data and observed data, respectively, and *θ *represents the unknown parameters. The MCMC method is conducted as follows: replace Y_*mis *_by some assumed values, then simulate *θ *from the resulting complete data posterior distribution P(*θ|Y*_*obs*_,*Y*_*mis*_). Let *θ*^(^^*t*^^) ^be the current simulated value of *θ*, then ${\text{Y}}_{\text{mis}}^{\text{(t}+\text{1)}}$ can be drawn from the conditional predictive distribution ${\text{Y}}_{mis}^{\left(t+1\right)}\sim \text{P}(Y_{mis}|Y_{obs},\theta^{\left(t\right)})$. Conditioning on ${\text{Y}}_{\text{mis}}^{\text{(t}+\text{1)}}$, the next simulated value of *θ *can be drawn from its complete data posterior distribution $\theta^{\left(\text{t}+1\right)}\sim P(\theta |Y_{obs},Y_{mis}^{\left(t+1\right)})$. By repeating the above procedure, we can generate a Markov chain $\left\{(\theta^{\left(t\right)},Y_{mis}^{\left(t\right)}):t=1,\;2,\;\ldots \right\}$ which converges in distribution to P(*Y*_*mis*_,*θ|Y*_*obs*_). This method is attractive since it avoids complicated analytic calculation of the posterior distribution of *θ *and Y_*mis*_. However, the distribution convergence is an issue that researchers need to face. In addition, this method is based on the assumption of multivariate normality. When using it for imputing binary variables, the imputed values can be any real values. Most of the imputed values are between 0 and 1, some are out of this range. We round the imputed values to 0 if it is less than 0.5 and to 1 otherwise.

This multiple imputation method is implemented using MI procedure in SAS. We use a single chain and non-informative prior for all imputations, and expectation-maximization (EM) algorithm to find maximum likelihood estimates in parametric models for incomplete data and derive parameter estimates from a posterior mode. The iterations are considered to have converged when the change in the parameter estimates between iteration steps is less than 0.0001 for each parameter.

### 2.3. Within-cluster multiple imputation

Standard MI strategies are inappropriate for handling the missing data from CRTs due to the assumption of independent observations. For the within-cluster imputation, we carry out standard MI described above using logistic regression method, propensity score method, and MCMC method separately for each cluster. Thus, the missing values are imputed based on the observed data within the same cluster as the missing values. Given that subjects within the same cluster are more likely to be similar to each other than those from different clusters, within-cluster imputation can be seen as a strategy to impute the missing values to account for the intra-cluster correlation. These MI strategies are implemented using MI, MIANALYZE, GENMOD, and NLMIXED procedures in SAS.

### 2.4. Across-cluster multiple imputation

#### 2.4.1. Propensity score method

Compared to the standard multiple imputation using propensity score method, we added cluster as one of the covariates to obtain the propensity score for each observation. Consequently, patients within the same cluster are more likely to be categorized into the same propensity score stratum. Therefore, the intra-cluster correlation is taken into account when the ABB procedure is applied within each stratum to generate the imputed values for the missing data. This multiple imputation strategy is implemented using MI, MIANALYZE, GENMOD, and NLMIXED procedures in SAS.

#### 2.4.2. Random-effects logistic regression

Compared to the predictive model using standard logistic regression method, we assume the binary outcome is modeled by the random-effects logistic model:

$$\log\text{it}(\mathrm{Pr}(Y_{ijl}=1))=X_{ijl}\beta +U_{ij}$$

where *Y*_*ijl *_is the binary outcome of patient *l *in cluster *j *in the intervention group *i*; *X*_*ijl *_is the matrix of fully observed individual-level or cluster level covariates, $U_{ij}\sim N(0,\;\sigma_{B}^{2})$ represents the cluster-level random effect, and $\sigma_{B}^{2}$ represent the between-cluster variance. $\sigma_{B}^{2}$ can be estimated when fitting the random-effects logistic regression model using the observed outcome and covariates. The MI strategy using random-effects logistic regression method obtains the imputed values in three steps:

(1) Fit a random-effects logistic regression model as described above using the observed outcome and covariates.

(2) Based on the estimates for *β *and σ_*B *_obtained from step (1) and the associated covariance matrix, construct the posterior predictive distribution of these parameters.

(3) Fit a new random-effects logistic regression using the simulated parameters from the posterior predictive distribution and the observed covariates to obtain the imputed missing outcome.

The MI strategy using random-effects logistic regression takes into account the between cluster variance, which is ignored in the MI strategy using standard logistic regression, and therefore may be valid for imputing missing binary data in CRTs. We provide the SAS code for this method in Appendix A.

#### 2.4.3. Logistic regression with cluster as a fixed effect

Compared to the predictive model using standard logistic regression method, we add cluster as a fixed effect to account for clustering effect. This multiple imputation strategy is implemented using MI, MIANALYZE, GENMOD, and NLMIXED procedures in SAS.

## 3. Simulation study

### 3.1. Community hypertension assessment trial

The CHAT study was reported in detail elsewhere [23]. In brief, it was a cluster randomized controlled trial aimed at evaluating the effectiveness of pharmacy based blood pressure (BP) clinics led by peer health educators, with feedback to family physicians (FP) on the management and monitoring of BP among patients 65 years or older. The FP was the unit of randomization. Patients from the same FP received the same intervention. In total, 28 FPs participated in the study. Fourteen were randomly allocated to the intervention (pharmacy BP clinics) and 14 to the control group (no BP clinics offered). Fifty-five patients were randomly selected from each FP roster. Therefore, 1540 patients participated in the study. All eligible patients in both the intervention and control group received usual health service at their FP's office. Patients in the practices allocated to the intervention group were invited to visit the community BP clinics. Peer health educators assisted patients to measure their BP and review their cardiovascular risk factors. Research nurses conducted the baseline and end-of-trial (12 months after the randomization) audits of the health records of the 1540 patients who participated in the study. The primary outcome of the CHAT study was a binary outcome indicating whether the patient's BP was controlled or not at the end of the trial. Patient's BP was controlled if at the end of the trial, the systolic BP ≤ 140 mmHg and diastolic BP ≤ 90 mmHg for patient without diabetes or target organ damage, or the systolic BP ≤ 130 mmHg and diastolic BP ≤ 80 mmHg for patient with diabetes or target organ damage. Besides the intervention group, other predictors considered in this paper included age (continuous variable), sex (binary variable), diabetes at baseline (binary variable), heart disease at baseline (binary variable), and whether patients' BP were controlled at baseline (binary variable). At the end of the trial, 55% patients' BP were controlled. Without including any other predictors in the model, the treatment effects and their 95% confidence intervals (CI) estimated from the GEE and RE model were 1.14 (0.72, 1.80) and 1.10 (0.65, 1.86), respectively. The estimated ICC was 0.077. After adjustment for the above mentioned variables the treatment effects and their CIs estimated from GEE and RE model were 1.14 (0.76, 1.70) and 1.12 (0.72, 1.76), respectively. The estimated ICC was 0.055.

Since there are no missing data in the CHAT dataset, it provides us a convenient platform to design a simulation study to compare the imputed and the observed values and further investigate the performance of the different multiple imputation strategies under different missing data mechanisms and percentages of missingness.

### 3.2. Generating dataset with missing binary outcome

Using the CHAT study dataset, we investigated the performance of different MI strategies for missing binary outcome based on MCAR and CD mechanisms. Under the assumption of MCAR, we generated dataset with certain percentage of missing binary outcome, which indicates whether the BP was controlled or not at the end of the trial for each patient. The probability of missing for each patient was completely at random, i.e. the probability of missing did not depend on any observed or unobserved CHAT data. Under the assumption of CD missing, we considered sex, treatment group, whether patients' BP controlled or not at baseline, which were commonly associated with drop out in clinical trials and observational studies [24-26], were associated with the probability of missing. We further assumed that male patients were 1.2 times more likely to have missing outcome; patients allocated to the control group were 1.3 times more likely to have missing outcome; patients whose BP was not controlled at baseline were 1.4 times more likely to have missing outcome than patients whose BP were controlled at baseline.

### 3.3. Design of simulation study

First we compared the agreement between the values of the imputed outcome variable and the true values of the outcome variable using Kappa statistics. Kappa statistic is the most commonly used statistic for assessing the agreement between two observers or methods which take into account the fact that they will sometimes agree or disagree simply by chance [27]. It is calculated based on the difference between how much agreement is actually present compared to how much agreement would be expected to be present by chance alone. A Kappa of 1 indicates the perfect agreement, and 0 indicates agreement equivalent to chance. Kappa statistic has been widely used by researchers to evaluate the performance of different imputation techniques on imputing missing categorical data [28,29]. Second, under MCAR and CD missing, we compared the treatment effect estimates from the RE and GEE methods under the following scenarios: 1) exclude the missing values from the analysis, i.e. complete case analysis; 2) apply standard multiple imputation strategies which do not take the intra-cluster correlation into account; 3) apply the within-cluster imputation strategies; and 4) apply the across-cluster imputation strategies.

We designed the simulation study according to the following steps.

1) Generated 5%, 10%, 15%, 20%, 30% and 50% missing outcomes under both MCAR and CD missing assumption. These amounts of missingness were chosen to cover the range of possible missingness in practice [30].

2) Applied the above multiple imputation strategies to generate *m *= 5 datasets. According to Rubin, the relative efficiency of the MI does not increase much when generating more than 5 imputed datasets [11].

3) Calculated Kappa statistic to assess the agreement between the values of imputed outcome variable and the true values of the outcome variable.

4) Obtained the single treatment effect estimate by combining the effect estimates from the 5 imputed datasets using GEE and RE model.

5) Repeated the above four steps for 1000 times, i.e. take 1000 simulation runs.

6) Calculated the overall Kappa statistic by averaging the Kappa statistic from the 1000 simulation runs. Calculated the overall treatment effect and its standard error by averaging the treatment effects and their standard errors from the 1000 simulation runs.

## 4. Results

### 4.1. Results when data are missing completely at random

With 5%, 10%, 15%, 20%, 30% or 50% percentage of missingness under MCAR assumption, the estimated Kappa for all different imputation strategies are slightly over 0.95, 0.90, 0.85, 0.80, 0.70, and 0.50 respectively. The estimated Kappa for different imputation strategies at different percentage of missing outcomes under the assumption of MCAR are presented in detail in Table 1.

**Table 1:** Kappa statistics for different imputation strategies when missingness is completely at random

Imputation level	Imputation strategies	Percentage of missingness					
		
		5%	10%	15%	20%	30%	50%
Within cluster	Logistic regression	0.954	0.913				
	
	Propensity score	0.953	0.910	0.865	0.820	0.730	0.549
	
	MCMC^1^	0.954	0.913	0.869	0.825	0.737	0.561

Across cluster	Propensity score	0.954	0.912	0.868	0.828	0.738	0.556
	
	Random-effects logistic regression	0.955	0.914	0.871	0.830	0.741	0.562
	
	Fixed-effects logistic regression	0.956	0.911	0.866	0.821	0.732	0.554

Ignore cluster	Logistic regression	0.954	0.907	0.861	0.814	0.722	0.537
	
	Propensity score	0.952	0.902	0.854	0.804	0.707	0.512
	
	MCMC^1^	0.953	0.906	0.859	0.811	0.717	0.530

Note:

1. MCMC = Markov chain Monte Carlo. For the MCMC methods, we round the imputed values to 1 if it is equal or greater than 0.5 and to 0 otherwise.

The estimated treatment effects and their 95% CIs obtained from different imputation strategies when missing is completely at random are presented in Table 2. For example, when 20% binary outcomes are MCAR and GEE model is used for analyzing the data, estimated treatment effects and the corresponding 95% CIs are 1.15 (0.76 1.72) from the complete case analysis, 1.15 (0.80 1.65) from the logistic regression method which ignores the cluster effect, 1.14 (0.73 1.77) from the within-cluster propensity score method, and 1.18 (0.80 1.74) from the across-cluster random-effects logistic regression method.

**Table 2:** Estimated treatment effects for different imputation strategies when missingness is completely at random

Imputation level	Imputation strategies	Analysis model	OR^4 ^and 95% CI^5 ^for Complete Data: GEE^2 ^1.14 (0.76 1.70) RE^3 ^1.12 (0.72 1.76)					
			
			OR^4 ^and 95% CI^5 ^for Different Percentage of missingness					
			
			5%	10%	15%	20%	30%	50%
Within cluster	Logistic regression	GEE^2^	1.14 (0.75 1.73)	1.14 (0.76 1.72)				
		
		RE^3^	1.13 (0.71 1.79)	1.13 (0.71 1.78)				
	
	Propensity score	GEE^2^	1.14 (0.75 1.74)	1.14 (0.75 1.73)	1.14 (0.74 1.75)	1.14 (0.73 1.77)	1.14 (0.72 1.82)	1.17 (0.68 2.01)
		
		RE^3^	1.12 (0.70 1.80)	1.12 (0.70 1.79)	1.12 (0.69 1.81)	1.12 (0.68 1.84)	1.12 (0.66 1.90)	1.14 (0.61 2.14)
	
	MCMC^1^	GEE^2^	1.14 (0.75 1.72)	1.13 (0.75 1.70)	1.13 (0.75 1.71)	1.12 (0.74 1.71)	1.12 (0.72 1.73)	1.11 (0.69 1.79)
		
		RE^3^	1.12 (0.71 1.78)	1.11 (0.70 1.76)	1.11 (0.70 1.77)	1.11 (0.69 1.78)	1.10 (0.67 1.79)	1.10 (0.64 1.87)

Across cluster	Propensity score	GEE^2^	1.14 (0.77 1.69)	1.14 (0.77 1.68)	1.14 (0.78 1.68)	1.14 (0.78 1.67)	1.15 (0.79 1.68)	1.16 (0.77 1.74)
		
		RE^3^	1.18 (0.88 1.59)	1.18 (0.88 1.59)	1.18 (0.87 1.60)	1.18 (0.87 1.61)	1.18 (0.85 1.64)	1.19 (0.80 1.77)
	
	Random-effects	GEE^2^	1.15 (0.78 1.69)	1.16 (0.79 1.70)	1.17 (0.80 1.72)	1.18 (0.80 1.74)	1.21 (0.80 1.81)	1.25 (0.75 2.06)
		
	logistic regression	RE^3^	1.14 (0.74 1.74)	1.15 (0.76 1.75)	1.17 (0.77 1.76)	1.18 (0.78 1.78)	1.21 (0.79 1.85)	1.25 (0.75 2.08)
	
	Fixed-effects	GEE^2^	1.14 (0.76 1.71)	1.15 (0.76 1.73)	1.15 (0.76 1.75)	1.16 (0.75 1.78)	1.16 (0.74 1.84)	1.18 (0.69 2.01)
		
	Logistic regression	RE^4^	1.13 (0.72 1.77)	1.13 (0.72 1.79)	1.14 (0.72 1.82)	1.14 (0.71 1.84)	1.15 (0.69 1.91)	1.16 (0.63 2.13)

Ignore cluster	Logistic regression	GEE^2^	1.14 (0.78 1.68)	1.14 (0.79 1.66)	1.15 (0.80 1.65)	1.15 (0.80 1.65)	1.16 (0.82 1.64)	1.16 (0.82 1.65)
		
		RE^3^	1.13 (0.74 1.73)	1.14 (0.75 1.71)	1.14 (0.77 1.70)	1.15 (0.78 1.68)	1.16 (0.81 1.66)	1.16 (0.82 1.66)
	
	Propensity score	GEE^2^	1.14 (0.78 1.67)	1.14 (0.79 1.66)	1.15 (0.80 1.65)	1.15 (0.81 1.64)	1.15 (0.82 1.61)	1.16 (0.82 1.62)
		
		RE^3^	1.13 (0.74 1.72)	1.14 (0.76 1.70)	1.14 (0.77 1.68)	1.15 (0.79 1.67)	1.15 (0.81 1.64)	1.16 (0.82 1.63)
	
	MCMC^1^	GEE^2^	1.14 (0.78 1.68)	1.14 (0.78 1.66)	1.14 (0.79 1.65)	1.14 (0.80 1.63)	1.14 (0.81 1.61)	1.15 (0.83 1.59)
		
		RE^3^	1.13 (0.74 1.73)	1.13 (0.75 1.70)	1.14 (0.77 1.68)	1.14 (0.78 1.66)	1.14 (0.80 1.63)	1.15 (0.82 1.60)

Complete case analysis		GEE^2^	1.14 (0.76 1.70)	1.14 (0.76 1.71)	1.14 (0.76 1.72)	1.15 (0.76 1.72)	1.15 (0.76 1.75)	1.16 (0.74 1.81)
		
		RE^3^	1.12 (0.72 1.76)	1.13 (0.72 1.76)	1.13 (0.72 1.77)	1.13 (0.72 1.78)	1.14 (0.72 1.81)	1.15 (0.71 1.87)

Note:

1. MCMC = Markov chain Monte Carlo. For the MCMC methods, we round the imputed values to 1 if it is equal or greater than 0.5 and to 0 otherwise.

2. GEE = Generalized estimation equation method

3. RE = Random-effects logistic regression

4. OR = Odds ratio

5. CI = Confidence interval

### 4.2. Results when missingness is covariate dependent

**Figure 1:**
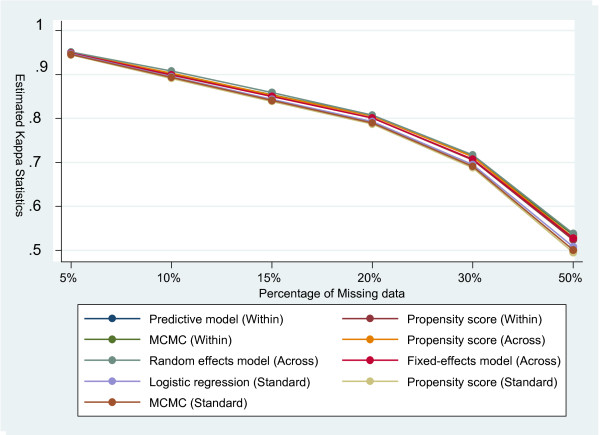
**Kappa statistics for different imputation strategies when missingness is covariate dependent**.

With 5%, 10%, 15%, 20%, 30% or 50% percentage of missingness under CD missing assumption, the estimated Kappa for all different imputation strategies are about 0.95, 0.90, 0.85, 0.80, 0.70, and 0.50 respectively. However, the estimated Kappa values are slightly less than those obtained under the MCAR assumption. The estimated Kappa values for different imputation strategies under the assumption of CD missing are presented in Table 3, and illustrated in Figure 1 in detail.

**Table 3:** Kappa statistics for different imputation strategies when missingness is covariate dependent

Imputation Level	Imputation strategies	Percentage of missingness					
		
		5%	10%	15%	20%	30%	50%
Within cluster	Logistic regression	0.949	0.902				
	
	Propensity score	0.947	0.899	0.850	0.801	0.706	0.524
	
	MCMC^1^	0.948	0.901	0.854	0.806	0.714	0.535

Across cluster	Propensity score	0.949	0.903	0.853	0.805	0.713	0.529
	
	Random-effects logistic regression	0.951	0.908	0.859	0.808	0.717	0.538
	
	Fixed-effects logistic regression	0.949	0.899	0.850	0.801	0.707	0.528

Ignore cluster	Logistic regression	0.947	0.895	0.844	0.793	0.695	0.508
	
	Propensity score	0.945	0.891	0.839	0.787	0.688	0.495
	
	MCMC^1^	0.946	0.893	0.841	0.790	0.691	0.501

Note:

1 MCMC = Markov chain Monte Carlo. For the MCMC methods, we round the imputed values to 1 if it is equal or greater than 0.5 and to 0 otherwise.

**Figure 2:**
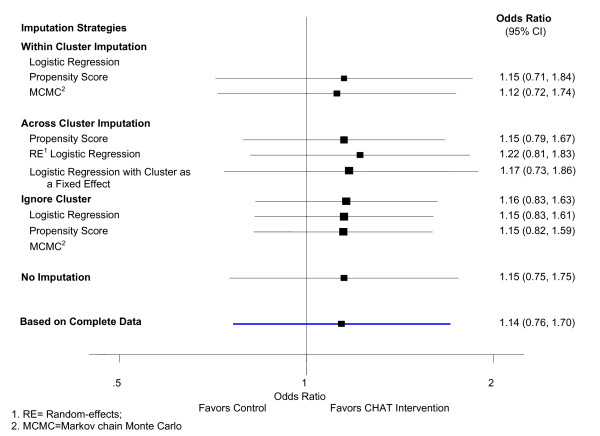
**Treatment effect estimated from generalized estimating equations when 30% data is covariate dependent missing**.

**Figure 3:**
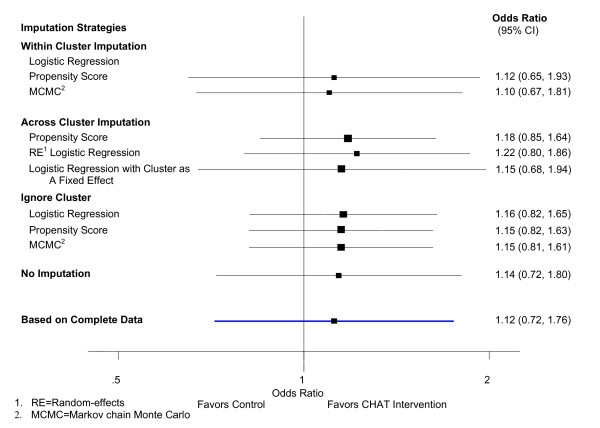
**Treatment effect estimated from random-effects logistic regression when 30% data is covariate dependent missing**.

The estimated treatment effects and their 95% CIs from GEE and RE model under the mechanism of CD missing are similar to those with MCAR mechanism as long as all the covariates associated with the missing data mechanism are adjusted for in the imputation. Details of the estimated treatment effects and their 95% CIs obtained from different imputation strategies when the missing iss covariate dependent are presented in Table 4, Figure 2, and Figure 3.

**Table 4:** Estimated treatment effects for different imputation strategies when missingness is covariate dependent

Imputation level	Imputation strategies	Analysis model	OR^4 ^and 95% CI^5 ^for Complete Data: GEE^2 ^1.14 (0.76 1.70) RE^3 ^1.12 (0.72 1.76)					
			
			OR^4 ^and 95% CI^5 ^for Different Percentage of missingness					
			
			5%	10%	15%	20%	30%	50%
Within cluster	Logistic regression	GEE^2^	1.14 (0.76 1.72)	1.14 (0.76 1.72)				
		
		RE^3^	1.12 (0.71 1.78)	1.13 (0.71 1.78)				
	
	Propensity score	GEE^2^	1.14 (0.75 1.72)	1.14 (0.75 1.73)	1.14 (0.74 1.75)	1.14 (0.73 1.78)	1.15 (0.71 1.84)	1.18 (0.68 2.04)
		
		RE^3^	1.12 (0.70 1.79)	1.12 (0.70 1.79)	1.12 (0.69 1.82)	1.12 (0.68 1.86)	1.12 (0.65 1.93)	1.15 (0.61 2.18)
	
	MCMC^1^	GEE^2^	1.13 (0.75 1.71)	1.13 (0.75 1.70)	1.13 (0.74 1.71)	1.12 (0.74 1.72)	1.12 (0.72 1.74)	1.12 (0.69 1.80)
		
		RE^3^	1.11 (0.70 1.77)	1.11 (0.70 1.76)	1.11 (0.69 1.77)	1.11 (0.69 1.78)	1.10 (0.67 1.81)	1.10 (0.64 1.88)

Across cluster	Propensity score	GEE^2^	1.14 (0.77 1.68)	1.14 (0.77 1.67)	1.14 (0.78 1.67)	1.14 (0.79 1.67)	1.15 (0.79 1.67)	1.15 (0.76 1.72)
		
		RE^3^	1.18 (0.88 1.59)	1.18 (0.87 1.59)	1.18 (0.87 1.60)	1.18 (0.86 1.61)	1.18 (0.85 1.64)	1.17 (0.78 1.76)
	
	Random-effects	GEE^2^	1.15 (0.78 1.69)	1.16 (0.80 1.70)	1.18 (0.81 1.72)	1.19 (0.81 1.75)	1.22 (0.81 1.83)	1.31 (0.83 2.06)
		
	logistic regression	RE^3^	1.14 (0.75 1.74)	1.16 (0.77 1.74)	1.18 (0.79 1.76)	1.19 (0.80 1.78)	1.22 (0.80 1.86)	1.31 (0.83 2.05)
	
	Fixed-effects	GEE^2^	1.14 (0.76 1.71)	1.15 (0.76 1.73)	1.15 (0.76 1.76)	1.16 (0.75 1.79)	1.17 (0.73 1.86)	1.17 (0.67 2.04)
		
	Logistic regression	RE^4^	1.13 (0.72 1.77)	1.14 (0.72 1.79)	1.14 (0.71 1.83)	1.15 (0.71 1.86)	1.15 (0.68 1.94)	1.15 (0.61 2.18)

Ignore cluster	Logistic regression	GEE^2^	1.14 (0.78 1.67)	1.14 (0.79 1.65)	1.15 (0.80 1.64)	1.15 (0.81 1.64)	1.16 (0.83 1.63)	1.15 (0.81 1.63)
		
		RE^3^	1.13 (0.74 1.72)	1.14 (0.76 1.70)	1.15 (0.78 1.68)	1.15 (0.80 1.67)	1.16 (0.82 1.65)	1.15 (0.81 1.63)
	
	Propensity score	GEE^2^	1.14 (0.78 1.67)	1.14 (0.79 1.65)	1.15 (0.81 1.64)	1.15 (0.82 1.63)	1.15 (0.83 1.61)	1.15 (0.82 1.62)
		
		RE^3^	1.13 (0.75 1.72)	1.14 (0.77 1.69)	1.15 (0.79 1.67)	1.15 (0.80 1.66)	1.15 (0.82 1.63)	1.15 (0.82 1.62)
	
	MCMC^1^	GEE^2^	1.14 (0.78 1.67)	1.14 (0.79 1.65)	1.15 (0.80 1.63)	1.15 (0.81 1.62)	1.15 (0.82 1.59)	1.13 (0.82 1.57)
		
		RE^3^	1.13 (0.74 1.72)	1.14 (0.77 1.69)	1.14 (0.78 1.67)	1.15 (0.80 1.65)	1.15 (0.81 1.61)	1.13 (0.82 1.57)

Complete case analysis		GEE^2^	1.14 (0.76 1.70)	1.14 (0.76 1.71)	1.14 (0.76 1.72)	1.15 (0.76 1.73)	1.15 (0.75 1.75)	1.15 (0.73 1.80)
		
		RE^3^	1.13 (0.72 1.75)	1.13 (0.72 1.76)	1.13 (0.72 1.77)	1.14 (0.72 1.78)	1.14 (0.72 1.80)	1.15 (0.71 1.85)

Note:

1. MCMC = Markov chain Monte Carlo. For MCMC methods, we round the imputed values to 1 if it is equal or greater than 0.5 and to 0 otherwise.

2. GEE = Generalized estimation equation method

3. RE = Random-effects logistic regression

4. OR = Odds ratio

5. CI = Confidence interval

## 5. Discussion

In this paper, under the assumption of MCAR and CD missing, we compared six MI strategies which account for the intra-cluster correlation for missing binary outcomes in CRTs with the standard imputation strategies and complete case analysis approach using a simulation study. Our results show that, first, when the percentage of missing data is low or intra-cluster correlation coefficient is small, different imputation strategies or complete case analysis approach generate quite similar results. Second, standard MI strategies, which do not take into account the intra-cluster correlation, underestimate the variance of the treatment effects. Therefore, they may lead to statistically significant but spurious conclusion when used to deal with the missing data from CRTs. Third, under the assumption of MCAR and CD missing, the point estimates (OR) are quite similar across different approaches to handle the missing data except for random-effects logistic regression MI strategy. Fourth, both within-cluster and across-cluster MI strategies take into account the intra-cluster correlation and provide much conservative treatment effect estimates compared to MI strategies which ignore the clustering effect. Fifth, within-cluster imputation strategies lead to wider CI than across-cluster imputation strategies, especially when the percentage of missingness is high. This may be because within-cluster imputation strategies only use a fraction of data, which leads to much variation of the estimated treatment effect. Sixth, larger estimated kappa, which indicates higher agreement between the imputed values and the observed values, is associated with better performance of MI strategies in terms of generating estimated treatment effect and 95% CI closer to those obtained from the complete CHAT dataset. Seventh, under the same imputation strategy and percentage of missingness, the estimates of the treatment effect from GEE and RE logistic regression models are similar.

To the best of our knowledge, limited work has been done on comparing different multiple imputation strategies for missing binary outcomes in CRTs. Taljaard *et al *[17] compared four MI strategies (pooled ABB, within-cluster ABB, standard regression, mixed-effects regression) for missing continuous outcome in CRTs when missing is completely at random. Their findings are similar to ours.

It should be noted that within-cluster MI strategies might only be applicable when the cluster size is sufficiently large and the percentage of missingness is relatively small. In the CHAT study, there were 55 patients in each cluster which provided enough data to carry out the within-cluster imputation strategies using propensity score and MCMC method. However, the logistic regression method failed when the percentage of missingness was high. This was because that when generating large percentage (≥20%) of missing outcome, all patients with binary outcome of "0" were simulated as missing for some clusters. Therefore, logistic regression model failed for these particular clusters. In addition, our results show that the complete case analysis approach performs relatively well even with 50% missing. We think that due to the intra-cluster correlation, one would not expect that the missing values have much impact if a large proportion of a cluster is still present. However, further investigation about this issue using a simulation study will be helpful to answer this question.

Our results show that the across-cluster random-effects logistic regression strategy leads to a potentially biased estimate, especially when the percentage of missingness is high. As we described in section 2.4.2, we assume the cluster-level random-effects follow normal distribution, i.e. $U_{ij}\sim N(0,\;\sigma_{B}^{2})$. Researchers have shown that misspecification of the distributional shape have little impact on the inferences about the fixed effects [31]. Incorrectly assuming the random effects distribution is independent of the cluster size may affect inferences about the intercept, but does not seriously impact inferences about the regression parameters. However, incorrectly assuming the random effects distribution is independent of covariates may seriously impact inferences about the regression parameters [32,33]. The mean of random effects distribution could be associated with a covariate, or the variance of random effects distribution could be associated with a covariate for our dataset, which might explain the potential bias from the across-cluster random-effects logistic regression strategy. In contrast, the imputation strategy of logistic regression with cluster as a fixed effect has better performance. However, it might only be applied when the cluster size is large enough to provide stable estimate for the cluster effect.

For multiple imputation, the overall variance of the estimated treatment effect consists of two parts: within imputation variance *U*, and between imputation variance *B*. The total variance *T *is calculated as *T *= *U *+ (1 + 1/*m*)*B*, where *m *is the number of imputed datasets [10]. Since standard MI strategies ignore the between cluster variance and fail to account for the intra-cluster correlation, the within imputation variance may be underestimated, which could lead to underestimation of the total variance and consequently the narrower confidence interval. In addition, the adequacy of standard MI strategies depends on the ICC. In our study, the ICC of the CHAT dataset is 0.055 and the cluster effect in the random-effects model is statistically significant.

Among the three imputation methods: predictive model (logistic regression method), propensity score method, and MCMC method, the latter is most popular method for multiple imputation of missing data and is the default method implemented in SAS. Although this method is widely used to impute binary and polytomous data, there are concerns about the consequences of violating the normality assumption. Experience has repeatedly shown that multiple imputation using MCMC method tends to be quite robust even when the real data depart from the multivariate normal distribution [20]. Therefore, when handling the missing binary or ordered categorical variables, it is acceptable to impute under a normality assumption and then round off the continuous imputed values to the nearest category. For example, the imputed values for the missing binary variable can be any real value rather than being restricted to 0 and 1. We rounded the imputed values so that values greater than or equal to 0.5 were set to 1, and values less than 0.5 were set to 0 [34]. Horton *et al *[35] showed that such rounding may produce biased estimates of proportions when the true proportion is near 0 or 1, but does well under most other conditions. The propensity score method is originally designed to impute the missing values on the response variables from the randomized experiment with repeated measures [21]. Since it uses only the covariate information associated with the missingness but ignores the correlation among variables, it may produce badly biased estimates of regression coefficients when data on predictor variables are missing. In addition, with small sample sizes and a relatively large number of propensity score groups, application of the ABB method is problematic, especially for binary variables. In this case, a modified version of ABB should be conducted [36].

There are some limitations that need to be acknowledged and addressed regarding the present study. First, the simulation study is based on a real dataset, which has a relatively large cluster size and small ICC. Further research should investigate the performance of different imputation strategies at different design settings. Second, the scenario of missing an entire cluster is not investigated in this paper. The proposed within-cluster and across-cluster MI strategies may not apply to this scenario. Third, we investigate the performance of different MI strategies assuming missing data mechanism of MCAR and CD missing. Therefore, results cannot be generalized to MAR or MNAR scenarios. Fourth, since the estimated treatment effects are similar under different imputation strategies, we only presented the OR and 95% CI for each simulation scenario. However, estimates of standardized bias and coverage would be more informative and would also provide a quantitative guideline to assess the adequacy of imputes [37].

## 6. Conclusions

When the percentage of missing data is low or intra-cluster correlation coefficient is small, different imputation strategies or complete case analysis approach generate quite similar results. When the percentage of missing data is high, standard MI strategies, which do not take into account the intra-cluster correlation, underestimate the variance of the treatment effect. Within-cluster and across-cluster MI strategies (except for the random-effects logistic regression MI strategy), which take the intra-cluster correlation into account, seem to be more appropriate to handle the missing outcome from CRTs. Under the same imputation strategy and percentage of missingness, the estimates of the treatment effect from GEE and RE logistic regression models are similar.

## Competing interests

The authors declare that they have no competing interests.

## Authors' contributions

JM conducted literature review, designed and implemented the simulation study, composed the initial draft of the manuscript. LT conceived the study. NAD, LD and LT provided consultation on matters of methodology and design. All authors reviewed and edited the manuscript before submission and provided assistance with the revision process.

## Appendix A: SAS code for across-cluster random-effects logistic regression method

%let maximum = 1000;

%macro parameter_estimate(percent,index);

   ods listing close;

   proc nlmixed data = mcar&percent&index cov;

      parms b0 = -0.0645 b_group = -0.1433 b_diabbase = -0.04 b_hdbase = 0.1224 b_age = -0.0066

         b_base_bpcontrolled = 1.1487 b_sex = 0.0873 s2u = 0.5;

      eta = b0 + b_group*group + b_diabbase*diabbase + b_hdbase*hdbase + b_age*age

         + b_base_bpcontrolled*base_bpcontrolled + b_sex*sex + u;

      expeta = exp(eta);

      p = expeta/(1+expeta);

      model outcome ~ binary(p);

      random u ~ normal(0,s2u) subject = assfpid;

      ods output ParameterEstimates = parameter&percent&index

         CovMatParmEst = covariance&percent&index;

   run;

   data parameter&percent&index;

   set parameter&percent&index;

      keep estimate;

   run;

   data covariance&percent&index;

   set covariance&percent&index;

      drop row parameter;

   run;

%mend parameter_estimate;

%macro mvn(percent, index, n);

   /* arguments for the macro:

   1. varcov: data set for variance-covariance matrix

   2. means: data set for mean vector

   3. n: sample size

   4. myMVN: output data set name */

   proc iml;

   use covariance&percent&index;/* read in data for variance-covariance matrix */

   read all into sigma;

   use parameter&percent&index;/* read in data for means */

   read all into mu;

   p = nrow(sigma);/* calculate number of variables */

   n = &n;

   l = t(half(sigma));/* calculate cholesky root of cov matrix */

   z = normal(j(p,&n,1234));/* generate nvars*samplesize normals */

   y = l*z;/* premultiply by cholesky root */

   yall = t(repeat(mu,1,&n)+y);/* add in the means */

   varnames = { b0 b_group b_diabbase b_hdbase b_age b_base_bpcontrolled b_sex s2u};

   create myMVN&percent&index from yall (|colname = varnames|);

   append from yall;

   quit;

%mend mvn;

%macro mi_random_effect(percent, index);

   %parameter_estimate(&percent, &index);

   %mvn(&percent, &index, 5);

   proc iml symsize = 512;

   use mymvn&percent&index;

   read all into mvndata;

   use mcar&percent&index;

   read all var {ptid DIABBASE HDBASE base_bpcontrolled last_bpimproved sex age assfpid_num group missing outcome} into temp_data;

   log_icca_cov = j(7700,12,0);

   do i = 0 to 4;

      do j = 1 to 1540;

         do k = 1 to 11;

            log_icca_cov[i*1540+j,k] = temp_data[j,k];

         end;

         log_icca_cov[i*1540+j,12] = i+1;

      end;

   end;

   do i = 1 to 7700;

      if log_icca_cov[i, 11] = . then do;

         num = log_icca_cov[i, 12];

         logit_p = mvndata[num, 1] + mvndata[num, 2]*log_icca_cov[i, 9]

            + mvndata[num, 3]*log_icca_cov[i, 2] + mvndata[num, 4]*log_icca_cov[i,3]

            + mvndata[num, 5]*log_icca_cov[i, 7] + mvndata[num, 6]*log_icca_cov[i,4]

            + mvndata[num, 7]*log_icca_cov[i, 6] + rand('NORMAL', 0, sqrt(mvndata[num, 8]));

         log_icca_cov[i, 11] = rand('BERNOULLI', exp(logit_p)/(1+exp(logit_p)));

      end;

   end;

   varnames = {ptid DIABBASE HDBASE base_bpcontrolled last_bpimproved sex age assfpid_num group missing outcome _imputation_};

   create log_icca_cov&percent&index from log_icca_cov (|colname = varnames|);

   append from log_icca_cov;

   quit;

%mend mi_random_effect;

%macro mi_icca_log(percent, index);

   ods listing close;

   %mi_random_effect(&percent, &index);

   data log_icca_cov&percent&index;

   set log_icca_cov&percent&index;

      if outcome > = 1 then outcome = 1;

      else if outcome < 1 then outcome = 0;

   run;

   proc freq data = log_icca_cov&percent&index;

   table last_bpimproved*outcome/kappa;

   ods output SimpleKappa = log_icca_kappapool&percent&index;

   run;

   data log_icca_kappapool&percent&index;

   set log_icca_kappapool&percent&index;

      if Label1 = 'Kappa';

   run;

   proc sort data = log_icca_cov&percent&index;

      by _imputation_;

   run;

   proc genmod data = log_icca_cov&percent&index;

      class outcome assfpid_num;

      model outcome = group diabbase hdbase age base_bpcontrolled sex/D = B link = logit;

      repeated subject = assfpid_num/type = exch covb;

      by _imputation_;

      ods output GEEEmpPEst = log_icca_geepar&percent&index

         GEERCov = log_icca_geecov&percent&index;

   run;

   data log_icca_geepar&percent&index;

   set log_icca_geepar&percent&index;

      if Parameter~ = 'Scale';

      if Parm = 'Prm' then Parm = 'Prm1';

      else if Parm = 'GROUP' then Parm = 'Prm2';

      else if Parm = 'DIABBASE' then Parm = 'Prm3';

      else if Parm = 'HDBASE' then Parm = 'Prm4';

      else if Parm = 'AGE' then Parm = 'Prm5';

      else if Parm = 'BASE_BPCONTROLLED' then Parm = 'Prm6';

      else if Parm = 'SEX' then Parm = 'Prm7';

   run;

   proc mianalyze parms = log_icca_geepar&percent&index covb = log_icca_geecov&percent&index;

   modeleffects Prm2;

   ods output ParameterEstimates = pool_log_icca_gee&percent&index;

   run;

   proc nlmixed data = log_icca_cov&percent&index cov;

      by _imputation_;

      parms b0 = -0.0645 b_group = -0.1433 b_diabbase = -0.04 b_hdbase = 0.1224 b_age = -0.0066

         b_base_bpcontrolled = 1.1487 b_sex = 0.0873 s2u = 0.5;

      eta = b0 + b_group*group + b_diabbase*diabbase + b_hdbase*hdbase + b_age*age

         + b_base_bpcontrolled*base_bpcontrolled + b_sex*sex + u;

      expeta = exp(eta);

      p = expeta/(1+expeta);

      model outcome ~ binary(p);

      random u ~ normal(0,s2u) subject = assfpid_num;

      ods output ParameterEstimates = log_icca_repar&percent&index

         CovMatParmEst = log_icca_recov&percent&index;

   run;

   proc mianalyze parms = log_icca_repar&percent&index covb = log_icca_recov&percent&index;

   modeleffects b_group;

   ods output ParameterEstimates = pool_log_icca_re&percent&index;

   run;

   ods listing;

%mend mi_icca_log;

%macro append_log_icca(percent);

   %do index = 1%to &maximum;

      %if &index = 1%then%do;

         data pool_log_icca_re&percent;

         set pool_log_icca_re&percent&index;

            run;

         data pool_log_icca_gee&percent;

         set pool_log_icca_gee&percent&index;

         run;

         data log_icca_kappa&percent;

         set log_icca_kappapool&percent&index;

         run;

      %end;

      %else%do;

         proc append base = pool_log_icca_re&percent data = pool_log_icca_re&percent&index;

         run;

         proc append base = pool_log_icca_gee&percent data = pool_log_icca_gee&percent&index;

         run;

         proc append base = log_icca_kappa&percent data = log_icca_kappapool&percent&index;

         run;

      %end;

   %end;

%mend append_log_icca;

%macro collect_result_log_icca(percent);

   %do index = 1%to &maximum;

      %mi_icca_log(&percent,&index);

   %end;

   %append_log_icca(&percent);

   proc univariate data = log_icca_kappa&percent;

      var nValue1;

   run;

   proc univariate data = pool_log_icca_gee&percent;

      var Estimate StdErr;

   run;

   proc univariate data = pool_log_icca_re&percent;

      var Estimate StdErr;

   run;

%mend collect_result_log_icca;

filename junk dummy;

proc printto log = junk;run;

%collect_result_log_icca(05);

%collect_result_log_icca(10);

%collect_result_log_icca(15);

%collect_result_log_icca(30);

%collect_result_log_icca(50);

proc printto; run;

## Pre-publication history

The pre-publication history for this paper can be accessed here:

http://www.biomedcentral.com/1471-2288/11/18/prepub
